# Toward Multiplexed Optogenetic Circuits

**DOI:** 10.3389/fbioe.2021.804563

**Published:** 2022-01-05

**Authors:** Ari Dwijayanti, Congqiang Zhang, Chueh Loo Poh, Thomas Lautier

**Affiliations:** ^1^ CNRS@CREATE, Singapore, Singapore; ^2^ Singapore Institute of Food and Biotechnology Innovation (SIFBI), Agency for Science, Technology and Research (A*STAR), Singapore, Singapore; ^3^ NUS Synthetic Biology for Clinical and Technological Innovation (SynCTI), Life Sciences Institute, National University of Singapore, Singapore, Singapore; ^4^ TBI, Université de Toulouse, CNRS, INRAE, INSA, Toulouse, France

**Keywords:** multiplexed regulation, optogenetic circuits, light-sensitive proteins, engineered photoreceptor modules, biotechnological applications, metabolic engineering

## Abstract

Owing to its ubiquity and easy availability in nature, light has been widely employed to control complex cellular behaviors. Light-sensitive proteins are the foundation to such diverse and multilevel adaptive regulations in a large range of organisms. Due to their remarkable properties and potential applications in engineered systems, exploration and engineering of natural light-sensitive proteins have significantly contributed to expand optogenetic toolboxes with tailor-made performances in synthetic genetic circuits. Progressively, more complex systems have been designed in which multiple photoreceptors, each sensing its dedicated wavelength, are combined to simultaneously coordinate cellular responses in a single cell. In this review, we highlight recent works and challenges on multiplexed optogenetic circuits in natural and engineered systems for a dynamic regulation breakthrough in biotechnological applications.

## Introduction

Living cells are able to sense, compute, and respond to changing conditions (Shah et al., 2006; Li et al., 2014b; Basan et al., 2015; Darlington et al., 2017; Patange et al., 2018). This adaptive strategy is enabled by coordinated interactions of cellular components in regulatory networks. In particular, a number of intra- and extracellular stimuli are perceived by a set of cellular sensor arrays. The internal stimuli rely on the change in the internal cellular state such as cellular burden and intracellular metabolites (Dahl et al., 2013; Ceroni et al., 2018), whereas the external stimuli sense environment signals including pH, oxygen, temperature, light, and small chemical inducer (Fernandez-Rodriguez et al., 2017; Moser et al., 2018; Meyer et al., 2019; Wang et al., 2021). Subsequently, these signals are further transmitted and potentially integrated to activate or repress complex cellular signaling cascades or genetic regulatory circuits. The output of this information processing will be further implemented in various cellular responses by actuators.

Both internal and external stimuli are key components to control gene expression and cell behavior in natural and engineered systems. In comparison to other stimuli, light-based systems are fast, non-invasive, and several are reversible, which is adapted for spatiotemporal control of gene expression. Typically, a photon is perceived by different light-sensitive proteins that subsequently change conformation. Each photoreceptor senses dedicated wavelengths to initiate spatiotemporal control of gene expression. Due to these inherent properties, a strong interest has emerged in exploring the use of light-sensitive proteins (Zayner and Sosnick, 2014). Recent reviews include photoreceptors in different organisms (Terakita, 2005; Grote and O’Malley, 2011; Bhaya, 2016; Liu et al., 2018), optogenetic regulation at the transcriptional level (Keyes and Mills, 2003; de Mena et al., 2018; Polesskaya et al., 2018; Baumschlager and Khammash, 2021), light-induced dimerization (Klewer and Wu, 2019), engineering strategies for optogenetics (McIsaac et al., 2015; Hoffmann et al., 2018; Bedbrook et al., 2019; Banerjee and Mitra, 2020), and application of optogenetics for bioproduction in which economical competitivity remains to be determined (Carrasco-López et al., 2020; Pouzet et al., 2020).

In nature, a set of native photoreceptors responsive to different wavelengths are found and governed to produce diverse physiological changes for robust light adaptation of particular organisms. This light-mediated multiplexed regulatory network has inspired researchers to employ multiple wavelengths as inputs in increasingly complex engineered systems (Tabor et al., 2011; Fernandez-Rodriguez et al., 2017). Nowadays, synthetic biology offers a range of modular tools to tune general cellular functions and/or plug synthetic pathways into endogenous metabolism. Since the multiplexed optogenetic circuits, using different wavelengths, allow for simultaneous control of cellular regulatory levels, it is possible to dynamically regulate the expression of a set of genes in synthetic genetic circuits. This multiplexed regulation would be desired as the complexity of synthetic genetic circuits increases (Moser et al., 2018). The multiplexed optogenetic circuits would leverage the design complexity of artificial cellular regulation and the number of regulated gene expressions compared to the use of a single light sensor in engineered systems. Importantly, the output response in the multiplexed optogenetic circuits can be easily combined and dynamically orchestrated through different light intensities and period of exposures (Ding et al., 2020; Lalwani et al., 2021).

This review provides a comprehensive overview focusing on light-driven multiplexed circuits that have not been specifically addressed in previous reviews. We first exemplified existing photoreceptors according to the detected wavelengths. We next described their spectral multiplexing in natural systems. Some notable examples of light-driven multiplexed regulation in the engineered system were also highlighted. Last, we discussed the current challenges and perspectives in implementing multiplex optogenetic circuits. Combining advantages of recent breakthroughs in optogenetics and genetic circuit design, multiplexed optogenetic circuits can become “plug-and-play” ways to create more sophisticated and robust regulation of engineered systems for various biotechnology and metabolic engineering applications.

## Light-Driven Control of Gene Expression and Cellular Activities

**FIGURE 1:**
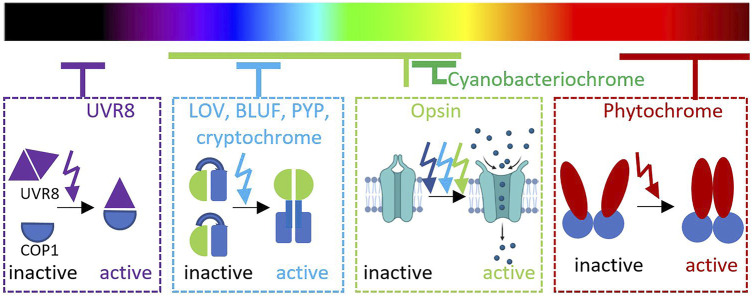
Distribution of photoreceptor families in different wavelengths.

The main source of light on Earth comes from the Sun that emits the entire spectrum of electromagnetic radiation. Most of the visible light can be absorbed and scattered through Earth’s atmosphere (Rayleigh, 1899). Spectrum and intensity of visible light illumination cause large temporal and spatial variation of light exposure that modulate light adaptation of organisms living in different biospheres (Endler, 1993; Tu et al., 2016; Ausprey, 2021). The ability to adapt to different levels of light exposure is particularly important for both photosynthetic and non-photosynthetic organisms. Organisms across kingdoms use light as one of the environmental signals in their adaptation such as in controlling motility, morphogenesis, immunity, stress response, and circadian rhythm (Hua, 2013; Jin and Zhu, 2019; Zhao et al., 2021b). Thus, various photoreceptors with different chromophores have evolved for different physiological adaptations by responding to different wavelengths and acting at different regulatory levels (Nagel et al., 2003; Guntas et al., 2015; Weber et al., 2019; Liao et al., 2020). Here, we exemplify representative photoreceptors in natural and engineered systems based on corresponding light perceived (Figure 1).

**GRAPHICAL ABSTRACT:**
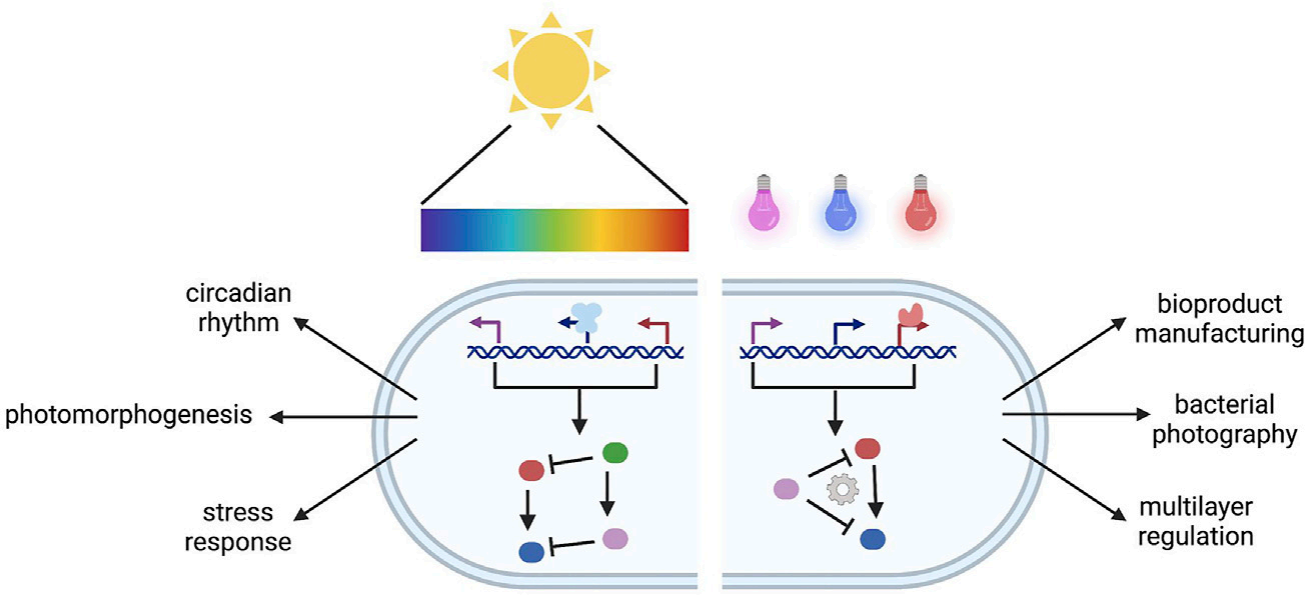
Natural and engineered multiplexing photoreceptors.

For an instance, UV-B light (280–315 nm) is absorbed by a Resistance Locus 8 (UVR8) photoreceptor to initiate signaling transduction of multiple mechanisms related to UV-B stress responses (Liang et al., 2019). This photoreceptor is constitutively expressed in the cytoplasm as a dimer in its inactive state. In *Arabidopsis thaliana*, three intrinsic tryptophan residues in the β-propeller core facilitate UV-B perception (O’Hara and Jenkins, 2012; Li et al., 2020). Upon UV-B light illumination, a dimeric UVR8 rapidly dissociates to accumulate two active monomers in the nucleus with the presence of COP1 (constitutively photomorphogenic 1) (Yin et al., 2016). The association of monomeric UVR8 and COP1 is a key regulation for photomorphogenesis, acclimatation, and tolerance to UV-B radiation, especially through hypocotyl growth suppression in *A. thaliana* (Favory et al., 2009; Cloix et al., 2012).

UV-A light with a longer wavelength (400 nm) is captured by a two-component signaling system, namely, UirS/UirR. This cyanobacteriochrome (CBCR) is naturally found in *Synechocystis* sp. PCC6803 for negative phototaxis (Song et al., 2011). As a CBCR member, UirS has two Per–Arnt–Sim (PAS) domains and a cGMP, adenylyl cyclase, FhlA (GAF) domain that interacts with phycocyanobilin chromophore (Song et al., 2011). In this study, it is hypothesized that UirR is released from membrane-associated UirS/UirR complex upon UV-A illumination. The free UiR then binds to the lsiR promoter and triggers the expression of LsiR. It should be noted that LsiR integrates inputs from multiple photosensors for directing the phototaxis and responds to other stress responses, such as ethylene (Kuchmina et al., 2017). Furthermore, Ramakrishnan and Tabor have exploited the versatility, fast dynamic, and photoreversibility of this UV–violet/green light switchable CBCR in *E. coli* and predicted its potential compatibility in multiplexed systems toward green/red and red/far red sensors (Ramakrishnan and Tabor, 2016).

Visible light in the range of 400–600 nm can be absorbed by opsin families. Similar to G-protein–coupled receptors, opsin protein consists of seven transmembrane structures commonly found in the animal retina (Terakita, 2005). A lysine residue in the seventh helix plays an important role as a retinal binding site. Photon absorption with an 11-*cis*-retinal chromophore by retinal triggers photoisomerization that results in conformational changes of the protein moiety and G-protein activation. Opsins can generally be subdivided into visual and non-visual opsins. In vertebrates, visual opsins can be found in rod and cone cells (Kefalov, 2012). Meanwhile, a type of non-visual opsin, namely, melanopsin is found in the skin of many vertebrates and is known to have an important role in circadian rhythm and other behavioral and physiological changes toward adaptation (Kelley and Davies, 2016).

Light-sensitive proteins with a similar structure to animal opsins are also found in bacteria, fungi, and algae. These photoreceptors function as light sensors or light-driven ion pumps. Rhodopsin-like protein as a photoreceptor was first found in the purple membrane of halophilic bacteria grown in high concentration of sodium chloride (Oesterhelt and Stoeckenius, 1971). Proteorhodopsin in marine bacteria is involved in the survival mechanism during the starvation condition (Gómez-Consarnau et al., 2010). The other microbial rhodopsin families, namely, channelrhodopsin-1 (ChR1) and channelrhodopsin-2 (ChR2) are first discovered in green alga *Chlamydomonas reinhardtii* (Nagel et al., 2002; Nagel et al., 2003). Due to their distinctive functions as light-gated cation channels, these light-sensitive proteins have been extensively studied and applied in the neuroscience field (Kleinlogel et al., 2011; Grossman et al., 2013; Ogren et al., 2014; Zabelskii et al., 2020). Importantly, the functionalization of channelrhodopsins (ChRs) in neuron cells is supported by the abundance of retinal chromophore in most vertebrate cells.

Due to its importance and potential application in neuroscience, a number of engineering strategies have been implemented to increase photocurrent, ion selectivity, kinetic, and wavelength sensitivity of ChRs (Lin, 2011; Bedbrook et al., 2019; Ku Cho et al., 2019). Berndt and others found that the original version of ChR2 expressed in mammalian cells has slow kinetics and small current, which limits its application in neuroscience (Berndt et al., 2011). Furthermore, random mutagenesis to create double E123T, T159C mutant of ChR2 has been shown to increase the photocurrent and faster kinetics compared to wild-type ChR2 (Berndt et al., 2011). Additionally, mutagenesis is also carried out to increase the selectivity of ChR2 toward calcium, allowing higher calcium permeability (Kleinlogel et al., 2011).

A wide range of wavelengths perceived by ChRs also allows for finding and generating a number of derivative ChRs corresponding to different lights. For example, a modification toward a blue-shifted ChR found in the alga *Scherffelia dubia* resulted in a ChR variant, namely, CheRiff that produces large photocurrent at 460-nm excitation (Hochbaum et al., 2014). The other ChR variants are found to be responsive toward red-shifted light such as VChR1 from *Volvox carteri* and *Ca*ChR1 from *Chlamydomonas augustae* (Zhang et al., 2008; Ogren et al., 2014). An improved version of red light–sensitive ChRs, namely, ReaChR (Lin et al., 2013) has been successfully combined with blue light–sensitive ChRs, that is, ChR2 to create independent dual-channel photostimulation in neurons (Hooks et al., 2015).

Blue light is strongly scattered throughout Earth’s atmosphere (Rayleigh, 1899). Due to its shorter wavelength and higher energy, blue light penetrates deeper in the deep sea to the dysphotic zone known as the twilight zone. These facts may contribute to the diversity of blue light–sensitive photoreceptors found in nature. The majority of natural photoreceptors use the light–oxygen–voltage (LOV) domain which is a subset of the PAS superfamily to sense blue light spectra (Krauss et al., 2009; Zoltowski et al., 2011; Losi et al., 2014). Typically, flavin adenine dinucleotide (FAD) or flavin mononucleotide (FMN) that is already available in abundance is used as a chromophore. Deriving from ancestral redox-active flavoproteins, the LOV photoreceptors harbor a conserved evolutionary structure and mechanism. Based on its mechanism in mediating signal transduction, the LOV domain can be classified into LOV1 and LOV2 domains (Crosson and Moffat, 2001). Molecular dynamic simulations indicated that destabilization of a highly conserved salt bridge activates LOV1, whereas a change in the flexibility of protein loops results in LOV2 activation (Freddolino et al., 2006). Furthermore, the LOV domain photocycle mechanism has been extensively studied in *Avena sativa* (*As*LOV2), emphasizing the key residue C450 in its reversible photocycle between blue light (488 nm) and dark conditions (Zayner and Sosnick, 2014).

**FIGURE 2:**
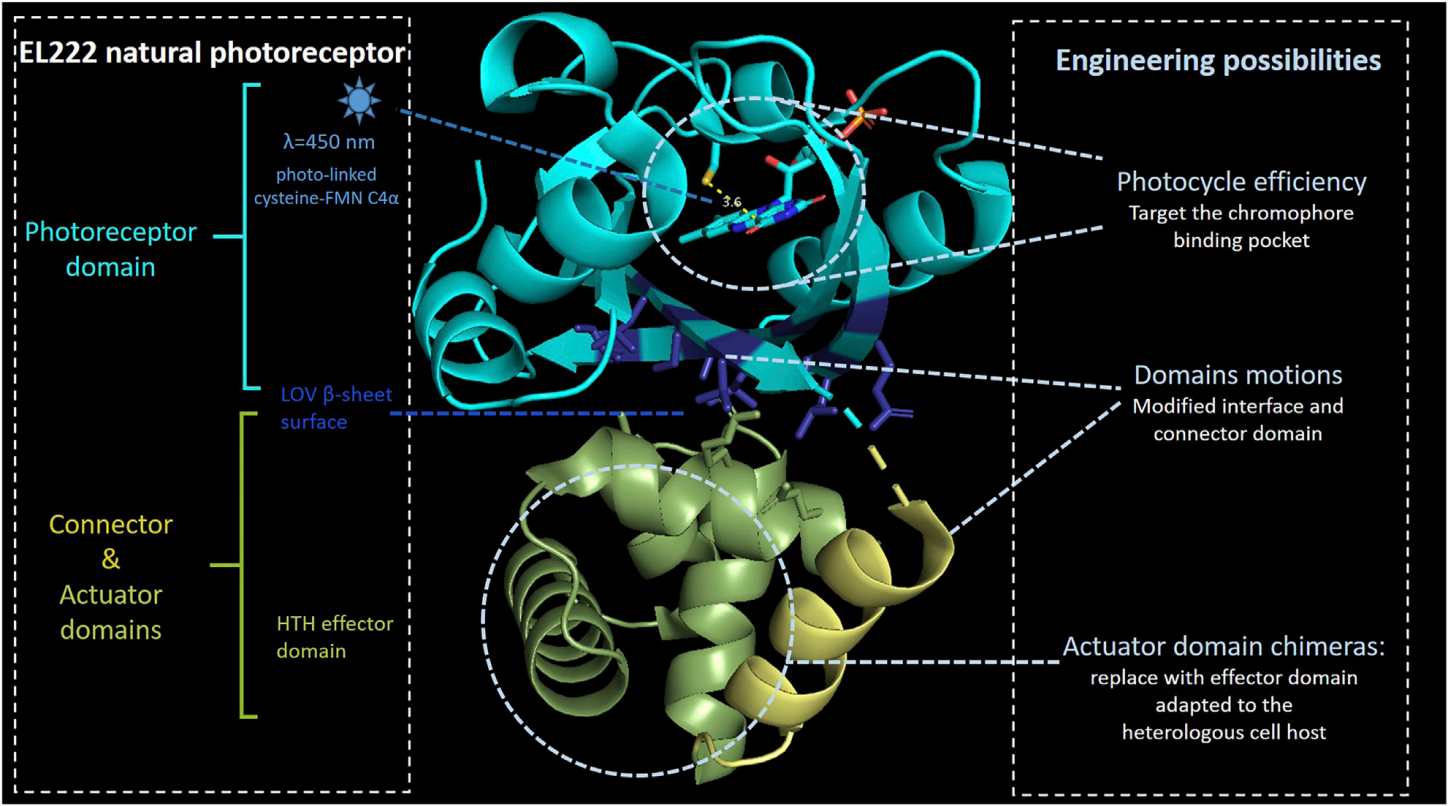
EL222 domains. Photosensing, connector, and actuator domains are, respectively, represented in light blue, gold, and green colors. FMN C4α distance with the sulfur atom of conserved cysteine is indicated in yellow. Under blue-light illumination, the FMN C4α coordinates with the cysteine 450 leading to a global domain motion, releasing the actuator domain to be active. The lateral chain of the β-sheet residues involved in the interface with the actuator domain is shown in dark blue. The model is based on the 3P7N pdb file.

The LOV domain is present in a wide range of light-sensitive proteins. One of them is blue light–regulated DNA-binding protein such as EL222, a 222–amino acid protein isolated from the marine bacterium *Erythrobacter litoralis* HTCC2594 (Rivera-Cancel et al., 2012; Zoltowski et al., 2013) (Figure 2). In addition to an N-terminal LOV domain, EL222 also contains a C-terminal helix–turn–helix (HTH) DNA-binding domain representative of LuxR-type DNA-binding proteins (Takakado et al., 2018). In an extensive approach (Nash et al., 2011), the EL222 structure has been solved in dark conditions, and NMR-established structure amplitudes have been obtained in illuminated conditions (pdb 3P7N). The dark-state crystal structure reveals that the conserved LOV β-sheet surface directly interacts with the 4α-helix and 1α-2α loop of the HTH domain, burying approximately 700 Å^2^ of surface area between the EL222 LOV and HTH domains, inhibiting HTH DNA-binding activity. Other key factor affecting the length of the photocycle is the electronic nature of the chromophore which plays a role in conformational change (as Q513 and N414 in AsLOV2). For example, N414 is not a conserved residue in LOV domains such as Vvd and YtvA, which have much slower photocycle times, 18,000 and 3,600 s, respectively, when compared to AsLOV2 (80 s) (Zayner and Sosnick, 2014). The EL222 originally role takes place at the transcriptional level through dimerisation processes upon blue light illumination. When the LOV domain absorbs blue light, it changes its conformation and exposes HTH domains to bind to a cognate DNA sequence (C20) (Rivera-Cancel et al., 2012) Beyond that, the small size of EL222 and abundance of cofactors could be beneficial for its portability in different hosts. Several studies have shown that the expression of EL222 and cognate promoters resulted in blue light–mediated transcriptional regulation (Jayaraman et al., 2016; Zhao et al., 2018; Ding et al., 2020; Zhao et al., 2021a). Jayaraman and others replaced the *lux* box in the native *luxI* promoter with the 18-bp EL222-binding region (Jayaraman et al., 2016). This promoter engineering yielded in a 5-fold transcription activation upon blue light illumination. Subsequently, a blue light–repressible promoter was constructed by positioning the EL222-binding region between −35 and −10 hexamers of the *E. coli* consensus promoter sequence so that the binding of RNAP is obstructed (Jayaraman et al., 2016). Since specificity and binding affinities of EL222 are mainly controlled by differences in the dissociation of DNA binding (Takakado et al., 2018), further improvement of the blue light–activated system has been done by extending the length of the EL222 region and increasing EL222 expression, whereas the fold repression of the blue light–repressible system has been optimized by changing the sequence of −35 and −10 hexamers surrounding the EL222-binding region (Ding et al., 2020). Additionally, EL222 has been fused to the viral VP16 transactivation domain to bind its corresponding promoter P_C120_ (Zhao et al., 2018). This system has further been shown to act as a blue light–mediated transcriptional regulator of synthetic metabolic pathways in *Saccharomyces cerevisiae*.

The LOV domain can also be found in VVD protein as the smallest blue light–responsive protein from filamentous ascomycetes such as *Neurospora crassa* (Schwerdtfeger and Linden, 2003). This photoreceptor can be implemented to control protein activity and localization in the reversible mode through dimerization of monomers in the presence of blue light. This small reversible photoreceptor has also been combined with LexA, a naturally found repressor protein in *E. coli* for DNA damage reparation, to create LEVI and LightOff gene expression system (Chen et al., 2016). During light illumination, a cysteine–flavin adduct is formed in the VVD domain and causes conformational changes of the domain. Subsequently, this mechanism will trigger dimerization of the fusion protein to bind its cognate operator sequence and repress promoter activity. Furthermore, optimization of the linker connecting LexA and VVD in LEVI resulted in 10,000-fold repression. Interestingly, the LightOff system has a comparable output as the T7-inducible system with lower leakage. Since LEVI consists of LexA, which regulates more than 20 genes responsible for DNA damage repair, there is a potential off-target effect. Therefore, a modification of the LexA-binding DNA moiety and cognate operator sequence was conducted to avoid the interference of endogenous LexA protein and operator region as in the bacterial SOS signal pathway (Chen et al., 2016). Other recent examples are a fusion of the VVD- and DNA-binding domain of AraC to create the BLADE system (Romano et al., 2021). This fusion protein regulates pBAD promoter using blue light illumination, instead of L-arabinose, which is known as its chemical inducer. The combination of VVD and transcriptional repressor TetR has also been shown to create a regulatory protein sensitive toward blue light and temperature (Dietler et al., 2021).

Furthermore, the LOV domain discovered in the N-terminal of the YtvA system from *Bacillus subtilis* plays an important role in coping with the stress environment through sigmaB-dependent stress response (Ávila-Pérez et al., 2006). Möglich and others swapped the heme-binding PAS domain of FixL from *Bradyrhizobium japonicum* with the LOV domain of *Bacillus subtilis* YtvA to create the YF1/FixJ system (Möglich et al., 2009). As a result, the kinase activity in the YF1/FixJ system is regulated by blue light illumination, instead of oxygen. Despite this, peptide linker which is used to fuse the natural light–sensitive proteins and effector has been found to significantly contribute to modulating the YF1/FixJ performance.

In addition to regulate gene expression at the transcriptional level, the LOV domain together with PAS and ANTAR domain has been demonstrated to regulate gene expression at the posttranscriptional level in PAL photoreceptor of *Nakamurella multipartite* (Weber et al., 2019). Blue-light illumination triggers the PAL photoreceptor to bind specifically to short RNA stem-loops and lower translation activity. Upon dark condition, the PAL protein is released from the aptamer, and translation is resumed. This posttranscriptional regulation via the LOV domain provides a great opportunity for multilevel regulation driven by blue light.

Another family of blue light–sensitive photoreceptors with flavin chromophore is blue light–using flavin (BLUF) domain (Park and Tame, 2017). Despite having the same cofactor as LOV domains, the BLUF domain has a unique photocycle mechanism due to its photoinduced proton-coupled electron transfer (Goings and Hammes-Schiffer, 2019). One of the most studied BLUF domains is AppA protein isolated from purple bacterium *Rhodobacter sphaeroides* (Masuda and Bauer, 2002). Interestingly, AppA protein interacts with a DNA-binding protein, namely, PpsR and acts as a transcriptional anti-repressor of genes related to photosynthesis by integrating redox and light signals (Braatsch et al., 2002).

While LOV and BLUF domains utilize FMN or FAD chromophores, one of the blue light–sensitive proteins, namely, photoactive yellow protein (PYP) domain covalently binds to p-coumaric acid chromophores (Van Der Horst et al., 2007). The PYP-containing PAS domain is originally isolated from halophilic phototrophic bacteria (Cusanovich and Meyer, 2003; Imamoto and Kataoka, 2007). Furthermore, the PYP domain is found in more than 140 species of bacteria and involved in a diverse functional roles such as phototaxis, cell buoyancy, DNA repair, and cyst formation (Meyer et al., 2012).

UV-A and blue light can also be sensed by cryptochromes (Cry): Cry1, Cry2, and Cry3. The majority of cryptochromes consist of an N-terminal photolyase-related (PHR) region and a cryptochrome C-terminal extension domain (Todo, 1999). Typically, these photoreceptors bind to pterin and flavin chromophores. Cry1 and Cry2, mainly located in the nucleus, are important for de-etiolation, flowering time, and circadian clock in *A. thaliana* (Tóth et al., 2001). Cryptochrome isolated from *A. thaliana* is identified to interact with basic helix–loop–helix 1, namely, Cry2/CIB1 (Liu et al., 2008). The Cry2/CIB1 system has been widely exploited in a number of optogenetic systems, especially in mammalian cells for intracellular signaling and subcellular organization (Duan et al., 2017; Benedetti et al., 2020). Similar to those in plants, cry1 and cry2 proteins play an important role in the generation and maintenance of mammalian circadian rhythm (Griffin et al., 1999).

One of the well-characterized membrane-associated blue light receptor kinases in *A. thaliana* is phototropins. Interestingly, light not only triggers phototropin activities but also impacts their expression level during plant development (Łabuz et al., 2012). These photoreceptors regulate phototropism, chloroplast positioning, and stomatal opening in *A. thaliana* (Rusaczonek et al., 2021). Phototropins consist of phot1 and phot2 with the PAS domain, specifically LOV1 and LOV2 in their N-terminal region to bind cofactor FMN (Christie et al., 2002).

Green light is well perceived through one of the well-studied cyanobacteriochromes in *Synechocystis* PCC6803 which is complementary chromatic acclimation (CCA) CcaS/CcaR two-component system. This light-sensitive protein is known to regulate the expression of the phycobilisome linker gene (cpcG2) (Hirose et al., 2008). The CcaS phosphorylates CcaR under green light illumination (535 nm) and activates the cpcG2 gene expression, whereas CcaR is dephosphorylated under red light illumination (672 nm) (Hirose et al., 2008).

With longer wavelength and low energy, red light is detected by several phytochromes containing PAS, GAF, and PHY domains. Phytochrome regulates a complex regulatory network in plant cells through transcriptional, translational, and posttranslational control including conformational switching and subcellular localization to promote morphogenesis, seed germination, de-etiolation, gravitropism, flowering time, and circadian clock (Cheng et al., 2021). The dimeric phytochrome is located in the cytoplasm and is covalently linked to a tetrapyrrolic cofactor (phycocyanobilin or phytochromobilin). The phytochrome dimer is then transported to the nucleus (Sakamoto and Nagatani, 1996), binds, and dimerizes the transcription factor PIF3 several times (Ni et al., 2013). These phosphorylations recruit ubiquitin ligases, polyubiquitinating the two proteins, a prelude to their rapid degradation by the proteasome (Ni et al., 2014; Ni et al., 2017). Light thus allows the control of the transcription factor PIF3. Note that even once entered the nucleus, phytochromes need light to bind to PIF3 (Ni et al., 1999). The isomerization of tetrapyrroles is reversible over time, and this reversion can be forced by illuminating the compound with far red light (∼640–720 nm) which isomerizes back the chromophores to their original state, leading to the inactivation of phytochromes (Gambetta and Lagarias, 2001). One of the phytochrome types found in fungal *Aspergillus nidulans* is FphA protein, which is important for sexual development and responsive toward red light (Blumenstein et al., 2005).

Instead of using the phycocyanobilin chromophore, a type of phytochrome found in bacteria, namely, bacteriophytochrome (BphP), uses incorporate biliverdin IXα (BV) tetrapyrrole (Piatkevich et al., 2013). BV is known to absorb most NIR light compared to other chromophores in phytochromes. Additionally, this chromophore is available in all mammalian cells (Chernov et al., 2017; Shcherbakova et al., 2018). Therefore, there has been a huge interest in engineering BphPs for application in mammalian cells supported by the characteristic of NIR light that could penetrate deeper into tissues (Chernov et al., 2017; Redchuk et al., 2018; Kaberniuk et al., 2021). One of the BphPs from the purple facultative photosynthetic bacteria *Rhodopseudomonas palustris* uses a bacteriophytochrome, namely, BphP1/PpsR2 system. Under NIR illumination, BphP1 changes its conformation and increases its affinity to bind toward PspR2 that subsequently derepresses photosynthetic gene expression (Braatsch et al., 2007).

Understanding the underlying mechanism of natural light–sensitive proteins combined with advanced strategies to engineer photoreceptors has served as a foundation to enable faster discovery and expansion of optogenetic toolboxes. Summary of the photoreceptors is provided in Table 1.

**TABLE 1:** Summary of photoreceptors across different taxa.

Photoreceptor	Wavelength (nm) on/off	Source organism	Type	Chromophore	Regulation	References
UVR8/COP-1	280–315	Plant, that is, *A. thaliana*	UVR8	Tryptophan	Posttranslation	Yin et al. (2016), Liang et al. (2019)
UirS/UirR	405/534	*Synechocystis* sp. PCC6803	Cyanobacteriochromes	Phycoviobilin	Transcription	Song et al. (2011); Ramakrishnan and Tabor (2016)
Opsin	400–600	Vertebrates and invertebrates	Opsin	11 *cis*-retinal	Posttranslation	Terakita (2005); Kefalov (2012)
Channelrhodopsin	400–600	*C. reinhardtii*	Opsin	all *trans*-retinal	Posttranslation	Nagel et al. (2002), Nagel et al. (2003)
CheRiff	460	*Scherffelia dubia*	Opsin	All *trans*-retinal	Posttranslation	Hochbaum et al. (2014)
VChR1	589	*Volvox carteri*	Opsin	All *trans*-retinal	Posttranslation	Zhang et al. (2008)
ReaChR	590–630	Modification from VChR1	Opsin	All *trans*-retinal	Posttranslation	Lin et al. (2013)
AsLOV2	450/dark	*A. sativa*	LOV	FMN	Transcription, posttranslation	Lungu et al. (2012); Zayner and Sosnick (2014)
EL222	450/dark	*Erythrobacter litoralis*	LOV	FMN	Transcription	Zoltowski et al. (2011), Zoltowski et al. (2013); Rivera-Cancel et al. (2012)
YtvA	450/dark	*B. subtilis*	LOV	FMN	Transcription	Losi et al. (2002), Gaidenko et al. (2006), Ávila-Pérez et al. (2006)
YtvA/FixJ	450/dark	*Bacillus subtilis* (YtvA) and *Bradyrhizobium japonicum* (FixL)	LOV	FMN	Transcription	Möglich et al. (2009)
Vivid (Vvd)	450/dark	*N. crassa*	LOV	FMN	Transcription	Schwerdtfeger and Linden (2003)
Magnet	450/dark	Modification from Vvd	LOV	FMN	Posttranslation	Kawano et al. (2015)
PAL receptor	465/dark	Nakamurella multipartita	LOV	FMN	Posttranscription	Weber et al. (2019)
AppA/PpsR	300–500	*Rhodobacter sphaeroides*	BLUF	FAD	Transcription	Braatsch et al. (2002); Masuda and Bauer (2002)
PYP	446	*Halorhodospira halophile*	PYP	p-Coumaric acid	Transcription	Cusanovich and Meyer (2003); Imamoto and Kataoka (2007)
Cry2/C1B1	450/dark	Plant, that is, *A. thaliana*	Cryptochrome	FAD	Transcription	Liu et al. (2008)
CcaS/CcaR	535/672	*Synechocystis* sp. PCC6803	Cyanobacteriochromes	Phycocyanobilin	Transcription	Hirose et al. (2008); Tabor et al. (2011)
FphA	707/754	*Aspergillus nidulans*	Phytochrome	Billin	Posttranslation	Blumenstein et al. (2005)
BphP1/PpsR2	760/640	*Rhodopsudomonas palustris*	Bacteriophytochrome	Biliverdin	Transcription	Braatsch et al. (2007)

## Light-Driven Multiplexed Regulation for Cellular Processes and Decision-Making

The number of available photoreceptors in a range of wavelengths possesses potential for their implementation in a more complex system such as multiplexed regulation. In particular, more than one photoreceptor with different wavelength sensitivities is expressed and synergistically combined in the same cells. This multiplexing mechanism is commonly found in nature, in particular in plants, and has recently been applied in engineered systems.

### Existing Light-Driven Multiplexed Regulation in Natural System

Nature provides a complex set of signals over the circadian cycle, including variation in temperature, light quality, and light quantity at varying rates of change (Millar, 2004). Several studies have focused on investigating individual light quality toward plant growth and development. The effect of different wavelengths has been widely assessed individually, through the determination of action spectra, establishing biological effectiveness as a function of the wavelength of incident light (Zavafer et al., 2015), although the majority of the system includes complex interaction with other wavelengths or even other inputs. Due to its properties for temporal dynamic and precise regulation, light-sensitive proteins compose different pathway architectures for signal propagation. In nature, genes encoding the major photoreceptors are not uniformly active throughout the day [reviewed in Fankhauser and Staiger (2002)]. Rather, the promoter activity of phytochromes and cryptochromes is diurnally regulated. Thus, a subset of photoreceptors act simultaneously to perceive different light attributes including duration, intensity, direction, and quality and result in specific regulatory system.

In plants, photoreceptor classes cooperate in the regulation of growth and other developmental processes such as phototropism and photoperiod. Each class, phytochromes, cryptochromes, and phototropins, are specific for a particular range of the visible spectrum and connected to overlapping signal transduction cascades. In general, angiosperms possess two cryptochromes, three or five phytochromes, and two phototropins, but special cases related to specific duplication events were occasionally observed (Lariguet and Dunand, 2005). For example, phototropism involves the integration of several types of light, to move forward or toward these stimuli. CRY1/phyA/phyB combines interference mechanisms for the regulation of auxin-responsive genes, leading to an asymmetric repartition of auxin, one of the major phytohormones. Under illumination on one side of the plant, auxin accumulates at the shaded face of the plant where it induces cell elongation, leading to bending the whole plant toward light (Gray, 2004). Plants use a number of photoreceptors to sense different wavelengths and subsequently respond to them through a complex signal cascade. Another example can be found in the adaptive regulation of phytohormones (Luo and Shi, 2019). These wavelength signals are integrated through a range of coexpressed photoreceptors: phytochromes, cryptochromes, ZTL, and UV-B photoreceptors. A cross talk between these receptors constitutes the light signaling pathway. In particular, UV-B photoreceptor UVR8 is combined with blue light–sensitive photoreceptor Cry1 to sequester the brassinosteroid signaling pathway (Liang et al., 2018; Wang et al., 2018).

**FIGURE 3:**
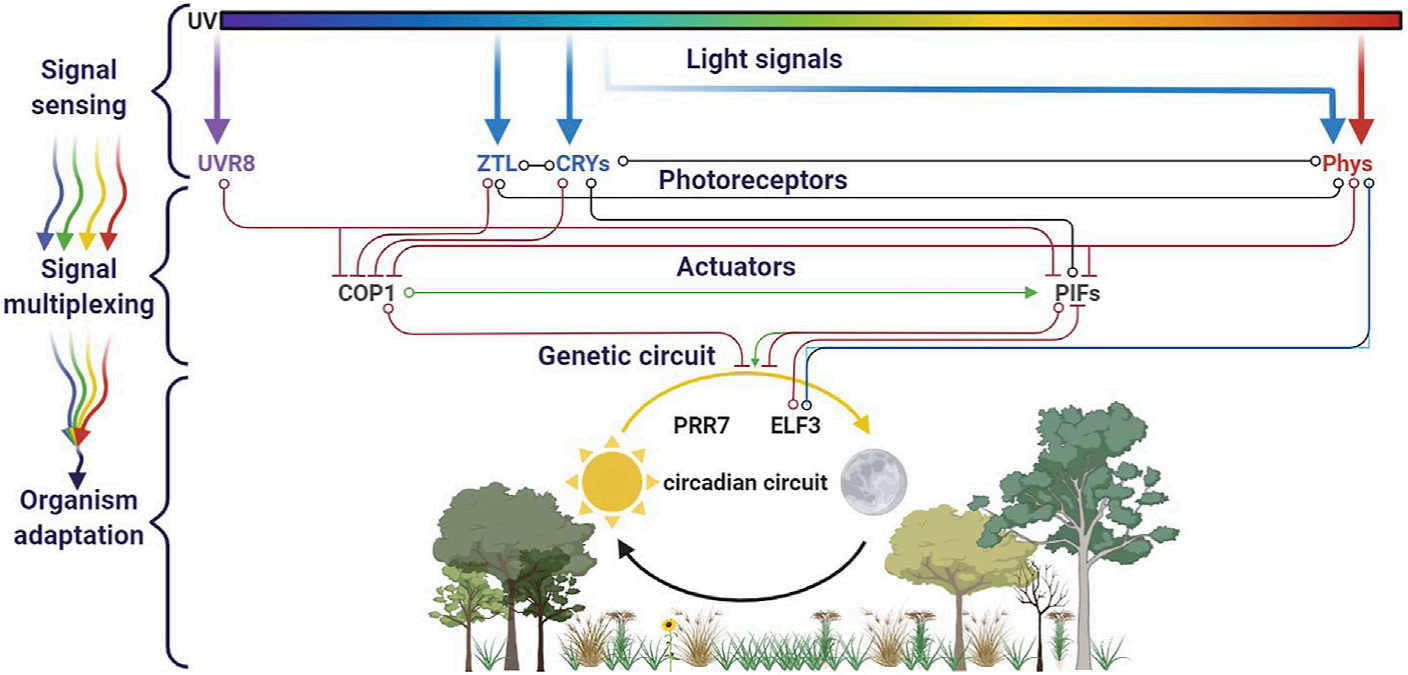
Circadian cycle and light multiplexing. Four families of photoreceptors (UVR8, ZTL, CRYs, and Phys) are involved in the plant circadian cycle. They cross-interact in a regulation network, in which branches evolve in function of the sun’s path. This figure was made using BioRender.

The *Arabidopsis* genome harbors five phytochrome genes (PhyA-E), two cryptochromes (Cry1 and 2), two phot family members (Phot1 and 2), three members of the LOV/F-box/Kelch proteins, and one UVR8. All these photoreceptors are not expressed simultaneously, but rather subgroups of photoreceptors are coexpressed in the function of the circadian cycle, such as PhYB with CRY1 at the dawn or later in the day PhyA with Cry2 (Tóth et al., 2001). This set of eleven photoreceptors allows a nearly whole light spectra integrative circadian regulation network (Figure 3). Additionally, several types of overlap between these receptors increase the linkages between the network branches. The first one involves the specificity and the quantity of a photoreceptor. In addition to red and far red light, blue light is known to activate PhyA and modulate phototropism (Lariguet and Fankhauser, 2004). Under very low-light conditions, PhyA accumulates to such high levels that its minor absorption of blue light causes significant circadian period shortening. Another cross talk involves a light-independent effect: cry1 is required for the wild-type response to low red light, although its absorption spectrum has no peak in red (Devlin and Kay, 2000). Additionally, to this intricate photoreceptor network, the multiplex also concerns the photoreceptors expressions themselves: The Phy and Cry photoreceptor genes are themselves targets of circadian regulation at the level of RNA abundance (Bognár et al., 1999; Hall et al., 2001; Toth et al., 2001).

Apart from the model plant *Arabidopsis*, the light transduction mechanism has also been investigated in food crops, that is, rice (*Oryza sativa*) (Lakshmanan et al., 2015). Using transcriptomic and metabolomic profiling, Lakshmanan and others found that blue and red lights facilitate the most divergent transcriptional responses that lead to distinguished plant phenotypes (Lakshmanan et al., 2015). Blue light can be absorbed by cryptochromes and phytochromes to upregulate photosynthesis and biosynthesis of secondary metabolites such as terpenoids and phenolic compounds. It is hypothesized that blue light also triggers the production of abscisic acid and represses ethylene biosynthesis that further inhibits stem elongation. In contrast, plant under red light treatment showed a significant reduction in photosynthesis and Calvin cycle. Red light absorbed by phytochromes also positively stimulates transcription of genes responsible for regulating specific ethylene signaling, hypocotyl elongation, and cell wall metabolism. Taken together, this study has demonstrated the multiplexed regulation of blue and red light in modulating plant growth and development.

Additionally, to combine independent photoreceptors inside one plant genetic circuit, nature has also built a multiplexing strategy using some original shortcuts. Neochromes, found in ferns, are natural chimeric photoreceptors in which phytochrome and phototropin modules are fused into a single protein (Li et al., 2014a). Hence, neochromes are able to use both red/far red and blue light to modulate phototropic responses.

Besides plant’s light sensing, animals and insects detect colors by intra- and intercellular multiplexing circuits, in the retinal cone cells. This ability comes from a required multicellular organization of several members of one type of photoreceptor family, the rhodopsins. Retina harbors three types of cone cells, expressing different opsins: L, M, and S opsins, named after their sensitivity in the long-, middle-, and short-wavelength regions of the spectrum. An intercellular level of multiplexing occurs by combining two main types of retinal ganglion cells: one targeting the activity of M and L photoreceptors, and the other one combining the activity of S and L + M photoreceptors. Another type of multiplexing appears at the intracellular level: cones coexpress different opsins. For example, in mice, most cones coexpress both S and M opsins in a common cone cell type throughout the retina (Applebury et al., 2000). Yellow color, for example, is perceived when the retinal L cones are stimulated slightly more than the M cones, and red color is perceived when the L cones are stimulated significantly more than the M cones. The *Drosophila* compound eye is formed from approximately 800 ommatidial units, a cluster of photoreceptor cells surrounded by support cells and pigment cells. Each ommatidium comprise six outer (R1–6) and two inner photoreceptors cells (R7 and R8). Photoreceptor cells express different rhodopsins as light molecular receptors. There are two types of ommatidia in the main part of the fruit fly eye: pale ommatidia express the short-UV–sensitive Rh3 rhodopsin in R7 and the blue-sensitive Rh5 in R8. Yellow ommatidia express the long-UV–sensitive Rh4 rhodopsin in R7 and the green-sensitive Rh6 in R8 (Yamaguchi et al., 2010). A general output signal multiplexing is based on intra-ommatidial interactions, comparing spectral information along with a UV versus visible axis, leading to insect sight (Heath et al., 2020).

Light multiplexing appears to be a widespread mechanism in nature, involved in different cell functions. The network complexity is based on the number of photoreceptors implicated, the possibility of an intermediate regulators layer, and a direct shortcut or feedback loop between sensors and actuators.

### Strategies to Multiplex Optogenetic Controls in Engineered Systems

**FIGURE 4:**
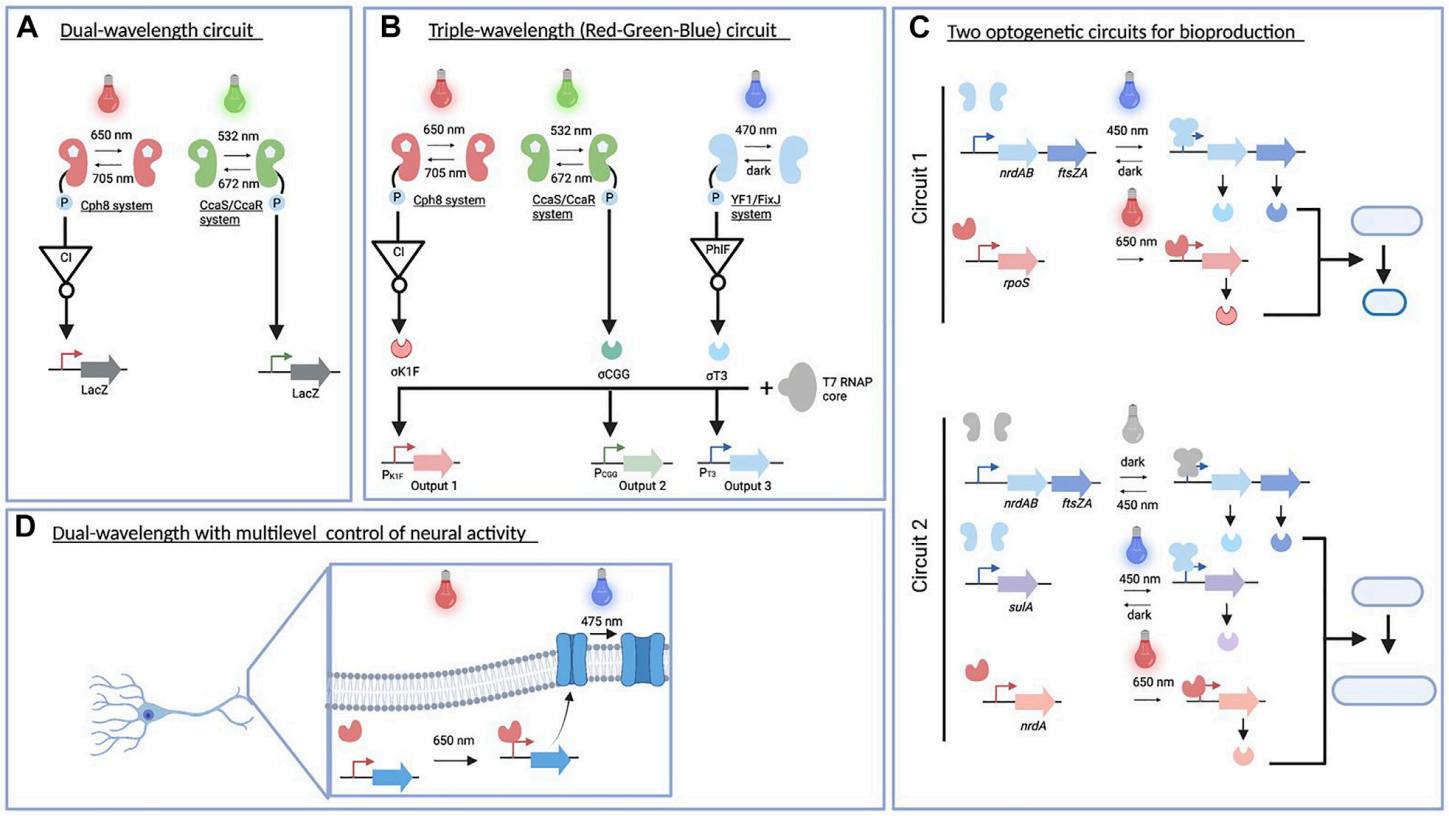
Multiplexed optogenetic circuits in engineered systems. **(A)**. The dual-wavelength optogenetic circuit is used for creating a bacterial photography (Tabor et al., 2011). **(B)** Red–green–blue circuit implemented for a colorful photography and modulated acetate production (Fernandez-Rodriguez et al., 2017) as well as biofilm patterning in diverse materials (Moser et al., 2019). **(C)** Two optogenetic circuits constructed for improving the production of acetoin and poly(lactate-co-3-hydroxybutyrate) (Ding et al., 2020). **(D)** Dual-wavelength controlling neural activity *in vivo* (Kaberniuk et al., 2021). This figure was made using BioRender.

Complex regulation of light-driven genetic circuits in natural systems has motivated researchers to subsequently develop multiplexed optogenetic circuits in an engineered system. This first requires the functional expression of light-sensitive proteins with an improved performance beyond their natural hosts. Historically, a proton pump rhodopsin (proteorhodopsin) from metagenomic mining of uncultured chemoautotroph marine gammaproteobacterium has been successfully expressed and resulted in photon translocation in *E. coli* upon light illumination (Beja et al., 2000). Subsequently, rhodopsin-based photoreceptors have also been shown to convert light into electrical activity in neuron cells and change animal behaviors, allowing for a non-invasive stimulation in neuroscience (Berndt et al., 2011; Deisseroth, 2015). Furthermore, this breakthrough has also inspired the engineering of diverse photoreceptors and their implementation beyond the field of neuroscience, including synthetic biology and metabolic engineering (Lalwani et al., 2018; Romano et al., 2021). Here, we exemplified some notable examples of multiplexed optogenetic applications, with a focus on microbial systems, that is, *E. coli* (Figure 4).

One of the first examples of an engineered multichromatic gene regulatory system in *E. coli* was published in 2011. Two-component systems driven by green/red light were functionally expressed to simultaneously regulate gene expression at the transcriptional level (Figure 4A) (Tabor et al., 2011). In particular, the green light sensor was built using the CcaS/CcaR system found in cyanobacteria (Hirose et al., 2008). The green light (535 nm) illumination optimally activates CcaS autophosphorylation and phototransfer to CcaR, leading to transcription from the P_*cpcG2*_ promoter. This process is reversed by red light (672 nm) absorption. On the other hand, the Cph8 red light sensor was constructed by fusing the effector histidine kinase domain EnvZ to the phytochrome Cph1, with active and inactive states in far red light (730 nm) and red light (672 nm) illumination (Levskaya et al., 2005). A genetic inverter of a CI repressor from phage λ was further introduced and expressed under the control of P_OmpC_ to create a red light–activated system. As both photoreceptors use a phycocyanobilin chromophore to create fully functional systems, this compound was supplied by heterologous expression of a two-gene operon (*pcyA* and *ho1*). Despite having a shared chromophore, each photoreceptor absorbs different wavelengths for maximum transcriptional outputs. In total, three different wavelengths were used to produce four different states. Both photoreceptors are reversible and orthogonal at a certain threshold of light intensity. By connecting both output promoters to the *lacZ* gene, the authors successfully generated a bacterial photography in a lawn of engineered cells harboring this optogenetic circuit and upon combination of green/red light illumination.

The previous success of multiplexed optogenetic circuits in creating bacterial photography (Tabor et al., 2011) has inspired Fernandez-Rodriguez et al. (2017) to expand the genetically encoded system that can respond to red, green, and blue (RGB) light spectra (Figure 4B) (Fernandez-Rodriguez et al., 2017). Following the previously established system, the red light–activated system was connected to the P _λ_ promoter, while the green light signal was used to trigger the transcription from the P_*cpcG2-172*_ promoter (Schmidl et al., 2014). In addition, a blue light sensor using a chimeric histidine kinase YF1 (Möglich et al., 2009) was deployed to sense a blue light signal (470 nm). This blue light photoreceptor uses s flavin mononucleotide chromophore that is active in the dark and inactive in response to blue light. To activate gene expression upon blue light illumination, a reverse design was implemented: the output promoter of the blue light sensor was used to drive the expression of a PhlF repressor. As a consequence, under blue light illumination, PhlF is no longer expressed, leading to a derepression of the genes under the control of the P_PhlF_ promoter. To modularize the output of signal processing, P_λ_, P_*cpcG2-172*_, and P_PhlF_ promoters were connected to a resource allocation system for transcription based on fragmented T7 RNA polymerase (Segall-Shapiro et al., 2014). Specifically, the P_λ_, P_*cpcG2-172*_, and P_PhlF_ promoters were used to drive the expression of sigma fragments containing DNA-binding domain, namely, σ_K1F_, σ_CGG_, and σ_T3_, respectively. The expression of these sigma factors coupled with constitutive expression of the core fragment enables the transcription of genes downstream the cognate P_K1F_ (red light), P_CGG_ (green light), and P_T3_ (blue light) promoters. To produce a colorful photograph, each of these promoters was used to generate colored pigments on a plate from specific enzymes, that is, glucuronidase (GusA), β-galactosidase (LacZ), and *Methylophaga* flavin-containing monooxygenase (bFMO). After 18 h of color image projection to RGB color strain spread in a plate, this RGB system resulted in a high-resolution color picture.

Linked to these proof of concepts, the multiplexed optogenetic circuit has been implemented to modulate the flux of a metabolic pathway such as acetate production (Fernandez-Rodriguez et al., 2017). The CRISPR interference (CRISPRi) system (Qi et al., 2013) was used to regulate three endogenous genes responsible for acetate production, namely, *pta*, *ackA*, and *poxB* genes. In this system, a catalytically inactivated dCas9 was constitutively produced in a low amount. Furthermore, they placed single-guided RNAs (sgRNAs) targeting each of these genes downstream the P_K1F_, P_CGG_, and P_T3_ promoters. As a result, individual and combination of red, green, and blue light exposure had a lower acetate production than RGB strain grown under dark conditions, despite having a comparable biomass accumulation.

Given that light provides precise spatiotemporal control in high resolution, the RGB circuit has also been demonstrated to advance pattern formation in diverse living materials by controlling cell adhesion and functionalization (Moser et al., 2019). It has been known that biofilm formation in *E. coli* is mainly produced through curli biogenesis expressed from two operons: *csgBAC* and *csgDEFG* (Chapman et al., 2002). The authors initially selected CgsA as a target since this enzyme is secreted in a soluble form in the extracellular medium and has been engineered to display peptide tags for increasing affinity of cell adhesion (Nguyen et al., 2014). To easily monitor optogenetic control of biofilm formation using specific antibodies labeling, three variants of CgsA were controlled by the RGB circuit: 1) CsgA without any tag from *csgBAC* operon was expressed under the P_T3_ promoter (blue light), 2) CsgA with HA affinity tag was placed downstream of the P_CGG_ promoter (green light), and 3) CsgA with a His affinity tag was controlled by the P_K1F_ (red light). Red, blue, and green light illumination to RGB strain over 18-h incubation resulted in cell attachment of engineered strains into a polystyrene plate, despite discrepancy in cell density for each color projection was observed. Furthermore, the versatility of this light-driven biofilm formation was shown in a range of materials including glass, mica, and 3D-printed plastic polymers. The engineered cells harboring the P_T3_-*csgBAC* operon and P_CGG_-GFP exhibited biofilm formation and GFP expression under the blue and green light illumination. They also showed that the engineered cells embedded in the biofilm could be combined with a chemical sensing system without significantly affecting the performance of the RGB circuit used in biofilm formation.

Taken together, these remarkable studies have highlighted diverse applications of multiplexed optogenetic circuits based on a two-component system to spatiotemporally control multigene expression in *E. coli*. However, the two-component system involves chromophore incorporation, conformational rearrangement, phosphosignaling, and phosphotransfer to its response regulator. This signaling process may require longer time to reach its steady state, need to additionally express chromophore genes, limit the number of regulated genes cloned in circuits, and consume cellular resources (Batchelor and Goulian, 2006; Fernandez-Rodriguez et al., 2017; Liu et al., 2018). To overcome these issues, a number of studies have combined photoreceptors from both one- and two-component systems in their multiplexed optogenetic circuits.

For example, two circuits (Figure 4C) responsive to blue and NIR light (Ding et al., 2020) composed of an engineered EL222 system (Jayaraman et al., 2016) and an engineered bacteriophytochrome diguanylate cyclase (Ryu and Gomelsky, 2014). These dual-wavelength optogenetic circuits controlled bacterial cell division, changed the cellular morphology of *E. coli*, and enhanced biochemical production (Ding et al., 2020). By placing genes critical to shortening and prolonging cell division under the control of multiplexed optogenetic circuits, their expressions were dynamically modulated throughout the cell cycle. The first circuit containing a blue and NIR light activation tool was used to specifically shorten cell division and enhance acetoin production. The expression of two genes responsible for shortening cell division, that is, *nrdAB* and *ftsZA* genes, was controlled under a blue light–activated system, while the expression of the *rpoS* gene that increases cell robustness was driven by the NIR light activation tool. Adjusting light intensity and switching time from blue light (450 nm) to NIR light (650 nm) successfully shortened cell division and increased acetoin production compared to those without an optogenetic circuit. The second circuit had a slightly different architecture in which *nrdAB* and *ftsZA* genes were expressed under blue light–repressible tool, whereas genes responsible for prolonging cell division such as *nrdA* and *sulA* genes were placed downstream NIR and blue light activation tools, respectively. This circuit was intended to prolong cell division and increase the poly(lactate-co-3-hydroxybutyrate) production. In the first phase of growth, the cell count was increased by expressing NrdAB and FtsZA under dark conditions. The engineered strain was subsequently exposed to blue and NIR light illumination to stimulate prolonging cell division. A further light intensity optimization of blue and NIR light illumination resulted in 2-fold improvement of poly(lactate-co-3-hydroxybutyrate) production. These multiplexed optogenetic circuits for regulating bacterial cell division, which include orthogonal (blue/red wavelengths) and overlapping (blue light for two opposite actuators) signals, may be applicable to improve the production efficiency of other high-value compounds.

All multiplexed optogenetic circuits presented before focus on transcriptional-based regulation using different light spectrs (Figure 4D). Recently, a multiplexed optogenetic circuit at transcriptional and posttranslational levels has been designed by combining a single-component NIR optogenetic system of *Idiomarina* sp. bacterial phytochromes known as iLight system and CheRiff channelrhodopsin (Kaberniuk et al., 2021). The combination of these photoreceptors exemplified multilevel and multiple wavelength regulation in the same cell as transcription of CheRiff channelrhodopsin in neurons was activated by iLight system upon red-light illumination (660 nm), whereas CheRiff activity was stimulated upon blue–green light exposure (475 nm). Since NIR light can be used in deep tissue, this multiplexed optogenetic regulation can be further applied to control neural activity *in vivo*.

## Challenges in Implementation of Multiplexed Optogenetic Circuits

The aforementioned optogenetic applications supported by the wide range of variants of optogenetic toolboxes have demonstrated the versatility of the system in increasingly complex genetic circuits. Despite this, the current optogenetic systems still have biological and physical challenges that may hamper their implementation. Current challenges for multiplexed optogenetic circuits are mainly in the selection of wavelength, choice of the photoreceptors to be combined, and circuit architecture.

### Combining Wavelengths in Living Cells

Light exposure for certain wavelengths can be harmful to living organisms. Damage occurs because light is radiant energy. This energy causes irreversible changes, either through radiant heating or photochemical action. A limitation of multiplexing could then come from light toxicity: combining several optogenetic wavelengths could increase light stress caused by overexcitation, with the emergence of reactive oxygen species (ROS) and the need to overexpress functioning of ROS-scavenging systems, as well as other protective mechanisms such as non-photochemical quenching. Moreover, UV and blue light can damage DNA through thymine cross-link, whereas infrared light brings locally non-required additional heat. These external stresses can force the cells to adapt by mutations, leading to strain genetic instability and source of bioprocess instability and of nightmares for the bioprocess engineers. It should be noted that some photoreceptors could be activated once and stay on its active state for some times. Then continuous lighting is not mandatory to trigger optogenetic regulation. However, some of them have a short active state due to the fast reversibility of the system. Therefore, the dose/length of light exposure needs to be modulated depending on the choice of photoreceptors. Spatial patterns of illumination have been simulated to dynamically regulate ChR2 expression in the neurons (Grossman et al., 2013). Temporal pulses in the light-duty cycle are sufficient to induce the optogenetic system while reducing the light exposure (Lalwani et al., 2021). Light pulsing has been implemented in engineered yeast harboring different metabolic pathways (Zhao et al., 2021a). Interestingly, the metabolite ratio is modified in the function of the light pattern, indicating that fine-tuning of the inducer can have a major effect on the actuator, opening room for more sustainable metabolic engineering design: do more and purest final product with less precursor. This exemplifies the need of a large-wavelength optogenetic toolbox and accurate light pattern design: what is the minimal light-duty cycle which enables maximum productivity? This light cycle will be specific to each engineered pathway and requires optimization from the microplate strain screening to scale-up in bioreactors. An original strategy could be at the cell level to mimic organelle motion observed in nature: the blue light receptor phototropin (phot) regulates intracellular chloroplast movements (Ishishita et al., 2020). At low illumination, chloroplasts accumulate to the cell surface to capture light efficiently (chloroplast accumulation response). If illumination increases too much, chloroplasts escape by moving to the side wall to reduce photodamage (chloroplast avoidance response).

At the single-cell level, light accurate delivery or, at the population level, homogenous penetration could be a physical challenge in the implementation of an optogenetic system. Temporal regulation of light pulses is possible at a scale inferior at 1 ms (Shemesh et al., 2017). Regarding spatial illumination control, mammal cell subcellular optogenetic regulation is possible (Guntas et al., 2015), but the equivalent level of precision obtained on-time regulation remains to be achieved in terms of spatial regulation, in order to control the intracellular mechanism differently in the function of the targeted cell area. Recently, the merging strategy, which combines the specificity and orthogonality of intrabodies with the spatiotemporal precision of optogenetics, has been developed (Redchuk et al., 2020). In mammalian cells, light stimulation is expected to deeply penetrate into tissues. However, only NIR light has the ability to deeply penetrate. To address the limitation issue with other wavelengths, the implementation of the light-based system in mammalian organisms is mediated by an internal optic fiber device (Deubner et al., 2019). As metabolite production in large bioreactors deals with dense cell populations, light diffusion could be problematic since each microbial cell may not receive simultaneously the same amount of light. This leads to population heterogenicity in the bioprocess, with asynchrony and unequal induction. Efforts are currently done in adapting the light-duty cycle itself, for example, by inverting the paradigm and using light to repress the metabolic pathway (Zhao et al., 2018). Therefore, at the beginning of the fermentation, the cell density is low, and light can diffuse more easily into the bioreactor, repressing the synthetic pathway, and the metabolic resources are fully used to produce biomass. When the biomass reaches the *ad hoc* density, the bioprocess is switched into the production stage. The dark cycle starts, the optogenetic actuators are not repressed, and enzymes of the synthetic pathway are expressed, leading to the metabolite of interest production. Nevertheless, this approach is less adapted for pulse design as at height cell density, light will be required for the inhibition part. To limit this issue, Zhao and others have recently created an OptoAMP circuit which amplifies the transcriptional response to blue light by as much as 23-fold compared to the basal circuit and allows efficient blue light activation of high–cell density culture in a 5-L bioreactor (Zhao et al., 2021a). Whatever the optogenetic design, at height cell density, each cell should receive the correct amount of light, according to the light-duty cycle. In a multiplexed design, an additional issue raises: each wavelength has a differential light penetration efficiency, leading to an increase in heterogenicity in the cell population: blue light penetration is weaker than the infrared one, leading to a less homogenous expression of the genes under the blue light stimulus. To tackle this limitation of light penetration and cell heterogenicity at a bioreactor scale, a new bioreactor architecture is required. In order that each cell encounters the required amount of photons, an optimization of stirring coupled with a dedicated internal light path device and/or external optogenetic loops is needed and, if possible, designed in a compatible way to be plugged into existing bioreactors (Pouzet et al., 2020).

Other properties to be taken into account in multiplexing will be the physical cross talk between different wavelengths with one photoreceptor. For example, it is known that *Arabidopsis* cryptochrome activation by blue light can be inhibited by green light *in vivo,* consistent with a change of the flavine cofactor redox state (Bouly et al., 2007). Combining the blue light sensor EL222 with the green light sensor CcaS/CcaR should then be thought with care for a useful cross-interaction. In the same view, multiplexing issues could come from other types of physical stimuli, for example, plant light sensor phytochromes also serve as temperature sensors through their thermal reversion capability. Temperature could then parasite the light effect. A library of *phyB* single mutants provides either thermal or light-independent behavior (Klose et al., 2020), highlighting the need to decipher each photocycle mechanism to engineer it.

### Photosensing Range, Actuator Response, and Core Component Expression

One photoreceptor limitation will be from the signal point of view: once light is sensed, the effector activity should be in a range compatible with its biological purpose. The initial performance of the opto-based system is often not comparable to that regulated by chemical inducers/repressors and requires a specific engineering technique to offer a better dynamic range. The photoreceptor initial low dynamic range and its improvement by engineering can be exemplified by the EL222-based system compared to the T7-based induction system (Lalwani et al., 2021). A slower performance has been shown in the CcaS/CcaR system compared to the trc-inducible system (Ariyanti et al., 2021). Rewiring a metabolic pathway with a low-induction system is an issue, and amplifier systems have been developed. For example, the dynamic range has been increased by tuning the component of opto-based regulation including by optimizing the binding site and the expression of the regulatory protein (Ding et al., 2020). With the same objective, the opto-T7RNAP design splits the polymerase into two fragments and fuses them into photoactivatable dimerization domains (Baumschlager et al., 2017). This system merges the dynamic of light regulation to the induction level of one of the most efficient promoters in bacteria, T7 promoter, leading to the amplification of initial light signal sensing. Nevertheless, this strategy is based on transcriptional regulation, with an inevitable lag between light sensing and the final effector activity. Despite this, the modification of the LEVI system has resulted in a comparable output to those with the T7 regulation system (Chen et al., 2016). Furthermore, some effort in using optogenetic systems for metabolic engineering approaches has demonstrated an improved yield production compared to that with conventional IPTG-induced systems (Lalwani et al., 2021). The corollary limitation is the output signal detection in multiplexing approaches. If we claim that light allows for a dynamic regulation, we need to quantify in real time an output signal, such as the production of metabolites. This output sensing enables feedback control to dynamically adjust the circuit. To achieve this real-time tuning, optogenetic actuators could be coupled in bioreactors with biosensors and enable *in vivo* metabolite sensing. Even if the type of biosensor needs to be diversified, recent studies have broadened the biosensor library, specific to the metabolic pathway of interest, such as monoterpenes (d’Oelsnitz et al., 2021) or phenol derivatives (Castaño-Cerezo et al., 2020). Connecting optogenetic systems to metabolite sensing has opened up cybergenetic applications [reviewed in Carrasco-López et al. (2020)]. A particular case is photosynthetic organisms. It is tempting to use light for both regulation and growth, that is, coupling synthetic pathway optogenetic regulation with autotrophic central metabolism through photosynthesis, as in microalgae. These organisms are already cultivated in photobioreactors, facilitating the implementation of light into the culture. Nevertheless, these “one-pot” light organisms will have to be engineered in terms of orthogonality of the optogenetic system to avoid cross talk with natural endogenous photoreceptors and light-harvesting complex of the photosystem.

Given that multiplexed optogenetic circuits need the coexpression of several photoreceptors, its implementation in different chassis could be hampered by their expressions themselves. Some designs require the expression of more than 20 genes, among them 14 genes are dedicated to the optogenetic core components, located into four plasmids (Moser et al., 2019). This could lead to metabolic burden and strain instability. Moreover, the majority of photoreceptors require specific cofactor, such as retinal or phycocyanobilin, which may not available in the chassis of interest. In some cases, a straightforward transferring photoreceptor from their natural hosts into a new host does not result in functional photoreceptors, especially for those with membrane-associated photoreceptors and two-component systems. Another example is CBCR/BphP which require bilin chromophores, not naturally produce in *E. coli*. Therefore, these hemic cofactors should be externally supplied or internally synthesized through heterologous expression of the heme pathway in *E. coli*. As FMN/FAD is abundantly available in *E. coli* and eukaryotic cells, the implementation of the one-component system such as LOV-based photoreceptors could directly be performed by expressing the photoreceptors (Kawano et al., 2015). In any case, it is often critical to validate that chromophores have been incorporated in the heterologous expressed apo-photoreceptor protein. Adding complexity, the cellular content of so many different regulators, for which the ratio balances the circuit equilibrium, could be asymmetrically spread through cell divisions and differential protein degradation, leading to an increase in cell population heterogenicity, harboring various contents of photoreceptors. Building genomic integrated optogenetic core components, in a set of synthetic chassis organisms, could help stabilize the core circuit expression and favor optogenetic circuit utilization into the scientist community.

### Circuit Architecture

The more photoreceptors and biosensors handled, the more genetic circuits will be intricated. Recent works start to implement several optogenetic systems into the same cell chassis. In these complex regulations, the difference of photocycle kinetics of each system will have to be taken into account to be coherent with this multilevel regulation. Multiplexing then could take two ways: 1) the orthogonal way: using different lights to trigger the corresponding sensors, 2) a dynamic way: take advantage of receptors sensing the same wavelength but harboring different photocycle periods (e.g., one fast dark dissociated coupled to a slow one). For example, incoherent feed-forward loop has been generated by combining the positive and negative regulations in yeast (Benzinger et al., 2021). This could lead to a unique type of light to sequential signal processing. Combining several photoreceptors, multiplexing could then be developed by designing intricated illumination patterns of several wavelengths, with one caveat. The photoreceptors are generally chosen in function of the signal perception of the lowest energy electronic transition (i.e., 445 nm for the FMN embedded in the LOV photoreceptor). Nevertheless, their secondary maxima may also play a very significant role (i.e., 355 nm for the FMN), leading to undesired activation when multiplexing the light sources to trigger other types of photoreceptors.

To expand dynamic regulation potential, it is possible to easily combine the photoreceptors to other known input sensors including chemical and temperature-sensing proteins to create more intricated regulatory circuits. Furthermore, the availability of optogenetic toolboxes could also potentially drive the exploration to other unwell explored physical sensors *in vivo*, such as radio wavelength, gravitropism, or magnetic fields, taking advantage of the *in vitro* progress in some of these fields (Yang and Lu, 2020). Orthogonal and hierarchical regulatory circuits will enable us to go toward fine-tunable synthetic organisms. By finding the real-time balance between endogenous chassis cell needs and heterologous pathway efficiency, engineered strains will harbor a intracellular sustainable architecture as a modular brick for sustainable bioeconomy.

## Conclusion

Adaptation ability toward light is supported by a number of photoreceptors. Discovery of light-sensitive proteins in nature has been the initial source of photoreceptors used in engineered biology. These photoreceptors are part of a multiplexing regulatory network, which enable living organisms to compile several external and internal stimuli. These light-multiplexed circuits, embedded in one cell, are based on 1) sensing several wavelengths, 2) interconnecting sensors with an intermediate regulatory layer, and 3) coordinating the actuator activity in the function of the compilated signals. Subsequentially, their performance and versatility have been significantly improved through engineering to expand the optogenetic toolbox in different synthetic biology hosts. In engineered systems, several photoreceptors have been combined, leading to a range of synthetic regulatory circuits. These circuits allow a fine and real-time tuning of targeted cell behaviors, opening up the road for dynamic metabolic control.

Taking advantage of already available engineered photoreceptors and synthetic circuits, multiplexing photoreceptors strategies still have to deal with wavelength combinations, *in vivo* compatibility, photoreceptor complementarity, and hierarchical circuit design using ingenious light pattern cycles. Future challenges remain to tackle such as tuning the kinetic behaviors of the natural and chimeric photoreceptors, simplifying the expression of the optogenetic core component, and implementing a fine real-time control with an autonomous back loop. Given the vast development with artificial intelligence of the protein structure and metabolism modeling, the next generation of robust photoreceptors could combine photoreceptor protein engineering to multiplexed regulation circuit design, in which a combination of photoreceptors, in an *ad hoc* and stable ratio, orchestrates the regulation of branched pathways into a synthetic microbial chassis. These improvements are anticipated to pave the way for an adoption of multiplexed regulation circuits in biotechnology and metabolic engineering applications.
